# Editorial: Translational strategies for chronic lung diseases: emerging therapies and precision medicine

**DOI:** 10.3389/fphar.2026.1940560

**Published:** 2026-10-07

**Authors:** Poonam Arora, Lalit Mohan Nainwal

**Affiliations:** 1 SGT College of Pharmacy, SGT University, Gurugram, Haryana, India; 2 Teerthanker Mahaveer College of Pharmacy, Teerthanker Mahaveer University, Moradabad, India

**Keywords:** artificial intelligence, chronic respiratory diseases, nextgen biological approaches, precision medicine, translational strategies

The increased incidence of chronic respiratory diseases, including COPD, asthma, idiopathic pulmonary fibrosis (IPF), and acute respiratory distress syndrome (ARDS), imposes a substantial burden not only on healthcare costs but also on the quality of life of patients (Calfee et al., 2014; Criner et al., 2019; Richeldi et al., 2014; King et al., 2014; Bhatt et al., 2023). Conventional pharmacological approaches are based on a “one-size-fits-all approach” and may be limited in terms of variable therapeutic responses, long-term safety, and associated side effects. Recent advancements in molecular biology, technology development, and medical science have substantially expanded the therapeutic landscape for chronic lung diseases (Yang et al., 2025). Emerging approaches involving next-generation biological strategies, including RNA interference, circular RNAs, microRNAs, short-hairpin RNAs, tissue regeneration, stem-cell-based therapies, and targeted drug delivery, are opening up new avenues for intervention that address disease mechanisms at a molecular level. Integration of these approaches with conventional pharmacotherapy may provide opportunities for more effective and targeted management of complex pulmonary diseases (Brewer et al., 2025).

Precision medicine has emerged as an important component of translational therapeutics (Wu et al., 2018). Advances in artificial intelligence and machine learning algorithms facilitate the interpretation of complex biomarker profiles and enable disease prediction for personalized treatment with high accuracy (Calle et al., 2014). Despite these advances, significant challenges remain in translating such technologies into routine clinical practice. These include biological complexity, safety, regulatory requirements, accessibility, and the need for robust clinical validation (Bakakos et al., 2025).

Against this background, the Research Topic “Translational Strategies for Chronic Lung Diseases: Emerging Therapies and Precision Medicine,” published in Frontiers in Pharmacology, brings together contemporary research that addresses the transition from experimental discoveries to possible meaningful applications with 17 contributions in total, including original research articles, case reports, systematic reviews, and comprehensive reviews, which collectively highlight recent advancements and existing gaps in the development of site-specific and targeted therapies. The contributions included in this Research Topic cover a broad spectrum of experimental research.

Studies on COPD explore molecular pathways such as RANKL–RANK signaling, artificial intelligence-based patient education, self-management, and the therapeutic potential of novel phosphodiesterase inhibitors such as ensifentrine. Zhou et al. demonstrated the association of RANKL–RANK signaling with increased MMP-9 expression in alveolar macrophages and CS-induced emphysema. Their study demonstrates the high translational potential of RANKL–RANK targets; however, broader implementation requires large-scale clinical studies to validate their effectiveness in comparison with standard interventions.

The role of hematological inflammatory biomarkers, including the neutrophil-to-albumin ratio, the neutrophil-to-lymphocyte ratio, and the eosinophil-to-lymphocyte ratio, was also examined for their potential clinical utility in predicting outcomes in chronic respiratory diseases. Wu et al. addressed the significant morbidity experienced by COPD patients and explained the development of an AI Health Education Accurately Linking System (AI-HEALS) to enhance COPD patients’ ability to self-manage and improve their quality of life. The translational potential of these systems is relatively high compared with preclinical discoveries; however, broader implementation requires validation across diverse patient populations. Almuntashiri et al., in a systematic review of randomized controlled clinical trials, reported significant improvements in the primary outcomes of test individuals treated with ensifentrine, presenting it as a mature therapeutic approach.

Asthma and inflammatory airway disorders were addressed through investigations into airway epithelial remodeling, immune responses, and phytochemical-based interventions. In particular, resveratrol was investigated by Chunxiao et al. for its potential molecular targets and regulatory effects on airway epithelial cells, mast cells, and eosinophils, highlighting the possibilities offered by bioactive compounds as complementary therapeutic candidates.

The Research Topic also emphasizes advances in diagnostic and monitoring technologies. Liquid biopsy approaches involving circulating tumor DNA, exosomal microRNA, and immune-cell populations were discussed as potential minimally invasive approaches for lung cancer detection and monitoring. Radiomultiomics, which combines quantitative CT scanning, multiomics, and AI methodologies, was recently applied to molecular phenotyping and endotyping of asthma (Zounemat Kermani et al., 2024). The presence of regulatory T cells, circulating tumor DNA, M2 macrophage polarization, and CD8^+^ T cells in BAL fluid could be considered real-time diagnostic measures for lung cancer. Imaging-based predictive models have moderate translational potential, particularly when based on clinically obtainable imaging data, subject to validation across populations and disease stages. In this context, the team led by Liu et al. presented a multiplanar reconstruction-based model to identify a novel imaging biomarker for pneumoconiosis screening.

Translational oncology is represented by reports on targeted and combination therapies for lung adenocarcinoma. The application of neoadjuvant targeted therapy, including osimertinib-based treatment, and combined chemotherapy, immunotherapy, and anti-angiogenic approaches illustrates the molecular characterization of tumors to convert unresectable lung adenocarcinoma to a resectable status, potentially improving clinical outcomes. Molecular targeted therapy is highly ready for translation and can be incorporated into precision oncology pathways.

Interventional bronchoscopy using laser, electrocautery, and cryotherapy further demonstrates the role of minimally invasive approaches in the management of selected thoracic malignancies. These bronchoscopic interventions represent clinically established or clinically advancing technologies, with considerably higher translational readiness than experimental molecular therapies. In a case report based on advanced (stage IIIA) lung adenocarcinoma with concurrent KRAS/G13C and TP53 mutations, Chen et al. suggested a prolonged combined regimen of platinum-doublet chemotherapy, immunotherapy, and anti-angiogenic therapy for better patient outcomes. This case report illustrates the potential of individualized treatment strategies and requires validation through larger clinical trials.

Natural and emerging therapeutic candidates are another important theme of this Research Topic. Experimental evidence regarding dipotassium glycyrrhizinate in ARDS and GTX-11 in fibrotic interstitial lung diseases highlights the continuing importance of identifying and translating novel therapeutic molecules into clinically relevant applications. Cao et al., in their experimental study, provided a foundation for the use of dipotassium glycyrrhizinate as a natural therapeutic agent to normalize the expression of various pro-inflammatory cytokines and free radical production, along with improved pulmonary histopathological damage in ARDS. A systematic review based on a compilation of case reports related to Qing-Hao-Bie-Jia Decoction, a representative Traditional Chinese Medicine (TCM) formula used to treat cancer-related fever, highlighted an improvement in the non-specific immune system as a preliminary therapeutic mode of action of the decoction. The study highlights existing gaps in research into the identification of active pharmacological ingredients.

Fibrotic interstitial lung diseases (ILDs) are characterized by inflammation and fibrosis of the lung parenchyma, leading to dyspnea, morbidity, and high mortality rates. Montes-Worboys et al. has suggested GTX-11 and its active metabolite, GTX-11m, as a possible therapeutic option for fibrotic ILD and pulmonary fibrosis in bleomycin-induced pulmonary fibrosis. Research examining glucose metabolism and its role in pulmonary inflammation further illustrates the growing recognition of metabolic pathways as potential therapeutic targets in chronic lung disorders. These findings provide proof-of-concept and support targeted drug development studies. Accumulating evidence reports that alterations in glucose metabolism play a crucial role in the development and progression of lung diseases under inflammatory conditions. The regulatory mechanisms of the metabolic and non-metabolic functions of glycolytic enzymes in various pulmonary disorders, in relation to disease pathogenesis, have been demonstrated to possess signaling capabilities and to exert regulatory effects on multiple key processes involved in immune cell activation and programmed cell death.

This Research Topic also considers sex-related differences in pulmonary arterial hypertension, environmental exposure to phthalates and their potential molecular effects, and therapeutic considerations in ventilator-associated pneumonia. Significant sex disparities have been observed in the prognosis of Pulmonary arterial hypertension (PAH), with worse outcomes typically experienced by men compared to women (Rodriguez-Arias and García-Álvarez, 2021). This disparity has been hypothesized to be due to differences in the expression and activity of androgen receptors. In this context, Sun et al. explained the functional role of androgen receptor inhibitors or gene knockout in attenuating pathological changes in PAH. The androgen receptor findings provide mechanistic evidence for a potential explanation of sex-related differences in PAH and identify a possible therapeutic pathway. This study therefore represents the discovery of early precision-medicine targets rather than a clinically validated therapy.

Given the widespread use of phthalates as plasticizers in polyvinyl chloride (PVC) products, including di(2-ethylhexyl) phthalate, diisononyl phthalate, and dioctyl phthalate, the article by Li et al. highlighted the potential contribution of phthalate exposure to the pathogenesis of respiratory diseases, particularly asthma and chronic obstructive pulmonary disease (COPD). Their findings have meaningful public health translational relevance, particularly with regard to preventive strategies.

A significant complication among critically ill patients receiving mechanical ventilation during prolonged intensive care and hospital stays is ventilator-associated pneumonia (VAP), which is associated with increased healthcare costs and mortality rates. In this context, Guan et al. investigated the association between dexmedetomidine (DEX) administration during ICU stays and mortality rates among patients with VAP. Because the study examined a clinically used drug in a real-world patient population, it had greater translational relevance than preclinical studies. These findings suggest that DEX use may be associated with improved clinical outcomes, highlighting its potential therapeutic role in the management of patients with ventilator-associated pneumonia.

Taken together, the contributions in this Research Topic demonstrate a broad and evolving translational landscape of chronic lung diseases, spanning molecular targets, emerging therapeutics, precision medicine, artificial intelligence, advanced diagnostics, environmental determinants, and clinically oriented interventions. Importantly, the evidentiary weight and translational readiness of these approaches vary considerably.

Their adoption requires reproducibility with clinically meaningful endpoints, assessment of safety and long-term outcomes, and integration into real-world healthcare settings. AI-enabled precision medicine and targeted therapies offer considerable potential for patient-centered care, but their broader implementation depends on robust clinical validation and accessibility.
